# The incretin/glucagon system as a target for pharmacotherapy of obesity

**DOI:** 10.1111/obr.13372

**Published:** 2021-10-28

**Authors:** Stefano Del Prato, Baptist Gallwitz, Jens Juul Holst, Juris J. Meier

**Affiliations:** ^1^ Department of Clinical and Experimental Medicine University of Pisa Pisa Italy; ^2^ Department of Internal Medicine IV Eberhard Karls University Tübingen Germany; ^3^ Institute for Diabetes Research and Metabolic Diseases of the Helmholtz Center Munich University of Tübingen Tübingen Germany; ^4^ Department of Biomedical Sciences, Faculty of Health and Medical Sciences University of Copenhagen Copenhagen Denmark; ^5^ Novo Nordisk Foundation Center for Basic Metabolic Research, Faculty of Health and Medical Sciences University of Copenhagen Copenhagen Denmark; ^6^ Division of Diabetology, Katholisches Klinikum Bochum, St. Josef Hospital Ruhr University Bochum Germany

**Keywords:** dual agonist, GLP‐1, glucagon, overweight

## Abstract

Obesity is a chronic, multifactorial, relapsing disease. Despite multicomponent lifestyle interventions, including pharmacotherapy, maintaining bodyweight loss is challenging for many people. The pathophysiology of obesity is complex, and currently approved pharmacotherapies only target a few of the many pathways involved; thus, single‐targeting agents have limited efficacy. Proglucagon‐derived peptides, glucagon, and the incretin hormones glucagon‐like peptide‐1 (GLP‐1) and glucose‐dependent insulinotropic polypeptide (GIP), represent attractive targets for managing obesity and metabolic disorders because they may have direct roles in multiple mechanisms including satiety, energy homeostasis, and lipolytic activity. Unimolecular dual and triple agonists targeting glucagon and incretin hormone receptors have been shown to promote bodyweight loss, lower glucose levels, and reduce food intake in animal models of obesity. Multiple dual receptor agonists are in clinical development for the treatment of obesity, including GLP‐1/GIP and GLP‐1/glucagon receptor agonists. The extent to which glucagon contributes to treatment effects remains to be understood, but it may promote bodyweight loss by reducing food intake, while concomitant GLP‐1 receptor agonism ensures normal glucose control. Further research is required to fully understand the molecular mechanisms of action and metabolic effects of both dual and triple receptor agonists.

## 1 INTRODUCTION

The overwhelming increase in the prevalence of obesity and people who are overweight in recent years represents one of the greatest global threats to public health. Worldwide, the prevalence of obesity has tripled since 1975, with over 650 million adults affected in 2016.^1^ Obesity is now recognized as a multifactorial disease, characterized by abnormal or excessive fat accumulation that presents a risk to human health.^2^ Obesity (body mass index [BMI] ≥30 kg/m^2^) and being overweight (BMI 25–29.9 kg/m^2^)^3^ are associated with several health conditions including diabetes, cardiovascular disease, some forms of cancer, musculoskeletal disorders (especially osteoarthritis), sleep apnea, asthma, gallstones, depression, and nonalcoholic steatohepatitis (NASH).^1^, ^2^, ^4^, ^5^, ^6^, ^7^ Obesity is a complex, chronic, relapsing disease; weight gain can be progressive, occurring over many years, and weight loss is difficult to achieve and even more difficult to maintain.^2^, ^8^, ^9^ In a meta‐analysis of 29 studies, more than half of lost weight (56%) was regained within 2 years and 79% of lost weight was regained by Year 5.^9^ Furthermore, some people with obesity do not consider themselves overweight, whereas others who do consider themselves overweight have no desire to lose weight.^10^ Around one third of people with obesity would like to lose weight but have not tried to do so within the past year and half have tried to lose weight without consulting a healthcare professional.^10^

### 1.1 Current treatment landscape

Current guidelines for obesity management recommend determining the degree to which an individual is overweight or has obesity and, depending on the severity, applying multicomponent interventions.^11^, ^12^, ^13^, ^14^, ^15^, ^16^ Lifestyle modifications are recommended for all patients who require weight loss, whereas additional pharmacotherapy is advised for individuals in whom lifestyle interventions have failed.^11^, ^12^, ^13^, ^14^, ^15^, ^16^ Lifestyle modifications can include reduced energy intake (typically to achieve an energy deficit of ≥500 kcal/day), increased aerobic physical activity levels to ≥150 min/week, and behavioral change strategies to facilitate adherence to diet and physical activity (self‐monitoring and reporting of dietary intake, physical activity, and weight measurements).^11^, ^12^, ^13^, ^14^, ^15^ A variety of diets designed to reduce energy intake may successfully result in weight loss in adults who are overweight or affected by obesity. Meal plans including Mediterranean‐style or vegetarian/vegan‐style diets, which are higher in plant‐based foods including olive oil (rich in monounsaturated oleic acid) and lower in processed food and meat than typical Western diets, may promote weight loss and cardiovascular benefits that are similar to those associated with low‐fat diets (25%–30% of calorie intake from fat).^11^, ^14^ Notably, in the Dietary Intervention‐Randomized Controlled Trial (DIRECT), a low‐fat diet in people with type 2 diabetes (T2DM) elicited a lower mean weight loss (2.9 kg) compared with a Mediterranean (4.4 kg) or a low‐carbohydrate (4.7 kg) diet.^17^ Compared with the low‐fat diet, the low‐carbohydrate diet improved lipid profiles, whereas the Mediterranean diet decreased fasting plasma glucose levels in patients with diabetes.^17^ A recent randomized controlled trial also showed that a 6‐week low‐carbohydrate diet, with high intake of protein and fat and energy intake adjustments to ensure weight stability, improved glycemic control and reduced liver fat content in patients with T2DM.^18^ These observations suggest that it is not necessarily fat intake that is responsible for increased fat deposition. Intermittent fasting has also gained interest for the treatment of obesity and diabetes, and has been recommended to comprise regular periods of no or very limited calorie intake (<25% of calorie requirement); for example, a 16‐h daily fast or a 24‐h fast on alternate days or two nonconsecutive days in a week.^19^ On nonfasting days, calorie intake can be unrestricted. A systematic review of 27 trials of people who were overweight or affected by obesity demonstrated that intermittent fasting reduces bodyweight by 0.8%–13% in the short term (2–52 weeks), regardless of change in calorie intake.^19^ In studies of patients with concurrent obesity and T2DM, improved glycemic control was also reported with intermittent fasting.^19^

With dietary interventions, most patients will reach a plateau in bodyweight loss at approximately 6–12 months, ranging from 3 to 12 kg, then will slowly regain weight over 2–5 years, with total weight loss reducing to 0 to 3–4 kg.^11^, ^12^ This pattern is most likely due to the progressive reduction of energy expenditure associated with bodyweight loss and the reduction of lean body mass. Therefore, long‐term bodyweight loss requires adjustment of lifestyle modifications over time. Adults who are unable to achieve or sustain bodyweight loss with comprehensive lifestyle modifications, who have either a BMI ≥30 or ≥27 kg/m^2^ with one or more comorbidities, can be considered for adjunct pharmacologic therapy.^11^, ^12^, ^13^

US Food and Drug Administration‐approved agents for the treatment of obesity include appetite suppressants, such as glucagon‐like peptide‐1 receptor (GLP‐1R) agonists (liraglutide and semaglutide), noradrenergic drugs (phentermine/topiramate and naltrexone/bupropion), and pancreatic lipase inhibitors (orlistat).^20^, ^21^ Phentermine stimulates noradrenaline release, which in turn suppresses appetite, augmented by topiramate, an anticonvulsant.^22^ Across randomized controlled trials, a mean bodyweight loss of 9.8 kg was observed with phentermine/topiramate treatment.^23^ Naltrexone acts as an opioid antagonist and bupropion as a dopamine and noradrenaline reuptake inhibitor, the combination of which promotes satiety and increased energy expenditure leading to a mean bodyweight loss of 4.4 kg.^23^, ^24^ Orlistat is a selective pancreatic lipase inhibitor that moderates intestinal absorption and digestion of fat, with an observed mean bodyweight loss of 3.1 kg.^22^, ^23^ A 2‐year study showed an additional bodyweight loss of ≥5% with the GLP‐1R agonist liraglutide, which was significantly greater, by 3.0 kg (*p* < 0.001), than weight loss with orlistat.^25^ In this trial, bodyweight loss stabilized by approximately 36 weeks,^25^ which was similar to that seen in trials of orlistat or the noradrenergic drug sibutramine.^26^, ^27^ Previous pharmacological agents approved for the treatment of obesity, including amphetamine derivatives, cannabinoid receptor blockers, and serotonin reuptake inhibitors, have been withdrawn due to their unfavorable adverse event (AE) profiles (Table 1).^22^, ^28^

**TABLE 1:** Previous pharmacological agents approved for the treatment of obesity and the AEs resulting in their withdrawal^28^, ^29^, ^30^

Agent	Mechanism of action	Launch date	Withdrawal date	Reason for withdrawal
Amfepramone (diethylpropion)	SNDRA	1957	1975	Cardiotoxicity
Amphetamine	SNDRA	1939	1973	Drug abuse/dependence
Aminorex fumarate	SRI	1962	1967	Cardiotoxicity
Benfluorex	SRI	1976	2009	Cardiotoxicity
Caffeine and ephedra	Nonselective adrenergic agonist	1994	2004	Cardiotoxicity, psychiatric
Chlorphentermine	SRI	1962	1969	Cardiotoxicity
Clobenzorex	SNDRA	1966	2000	Drug abuse, psychiatric
Cloforex	SRI	1965	1967	Cardiotoxicity
Cyclovalone + retinol + tiratricol	Bile acid secretion	1964	1988	Hepatotoxicity
Dexfenfluramine	SRI	1995	1997	Cardiotoxicity
Fenbutrazate	NDRA	1957	1969	Drug abuse, psychiatric
Fenfluramine	SRI	1973	1997	Cardiotoxicity
Fenproporex (perphoxene)	NRA	1966	1999	Drug abuse, psychiatric
Iodinated casein strophanthin	Thyroxine analogue	1944	1964	Endocrine, metabolism
Levoamphetamine	SNDRA	1944	1973	Drug abuse/dependence
Lorcaserin	Serotoninergic agonist	2012	2020	Increased risk of cancer
Mazindol	NDRA	1970	1987	Drug abuse, psychiatric (interaction with lithium)
Mefenorex (methylphenethylamine)	SNDRA	1966	1999	Drug abuse, psychiatric
Methamphetamine (desoxyephedrine)	SNDRA	1944	1973	Drug abuse/dependence
Phendimetrazine	NDRA	1961	1982	Drug abuse
Phenmetrazine	NDRA	1956	1982	Drug abuse
Phentermine^a^	NDRA	1959	1981	Drug abuse
Phenylpropanolamine (norpseudoephedrine)	NDRA	1947	1987	Hemorrhagic stroke
Pipradrol	NDRI	1953	1982	Drug abuse
Pyrovalerone	NDRA	1974	1979	Drug abuse
Rimonabant	Cannabinoid antagonist/inverse agonist	2006	2007	Psychiatric
Sibutramine	SNRI	2001	2002	Cardiotoxicity, psychiatric

Abbreviations: AE, adverse event; NDRA, noradrenaline–dopamine releasing agent; NDRI, noradrenaline–dopamine reuptake inhibitor; NRA, noradrenaline releasing agent; SNDRA, serotonin–noradrenaline–dopamine releasing agent; SNRI, serotonin–noradrenaline reuptake inhibitor; SRI, serotonin reuptake inhibitor.

^a^ Approved for use up to 12 weeks.

Bariatric surgery is an option for individuals with a BMI ≥40 kg/m^2^ or ≥35 kg/m^2^ and with comorbidities for which appropriate nonsurgical methods have failed.^11^, ^12^, ^13^, ^14^, ^15^, ^16^, ^31^ Roux‐en‐Y gastric bypass, often called gastric bypass, has traditionally been considered the gold standard bariatric procedure for weight loss. The underlying mechanisms are loss of appetite resulting in reduced food intake, most likely driven by the exaggerated secretion of gut hormones that occurs a few days after surgery. The increased secretion of these hormones, including glucagon‐like peptide‐1 (GLP‐1) and peptide YY (PYY), is due to accelerated exposure and absorption of nutrients in the small intestine.^32^, ^33^, ^34^ Changes in anatomy leading to mechanical restriction of food intake and malabsorption of macronutrients were originally thought to be responsible for weight loss following bariatric surgery. However, these effects have since been found to be inappreciable,^34^ except with less commonly used procedures such as jejunoileal bypass, biliopancreatic diversion, and duodenal switch, which dramatically reduce intestinal resorption of nutrients. The mode of action of gastric sleeve operations, now the most widely used procedure to treat obesity,^35^ is not fully elucidated, but the accelerated passage of nutrients into the small intestine, which also leads to exaggerated gut hormone secretion, is thought to play a role.^36^ Most surgical procedures are, in principle, irreversible and are not without complications^37^; moreover, surgical intervention alone is unlikely to manage obesity in the majority of patients. Therefore, there is a large unmet medical need for a highly efficacious pharmacological agent with a favorable benefit–risk profile for the treatment of obesity, especially in chronically ill patients with concomitant disease (e.g., hypertension, T2DM, and chronic obstructive pulmonary disease).

### 1.2 Rationale for targeting the incretin/glucagon system in obesity

Energy balance is maintained by an intricate network of interacting feedback mechanisms involving the hypothalamus, the brainstem, higher brain centers and, in the periphery, the stomach, gut, liver, thyroid, endocrine pancreas, and adipose (fat) tissue.^38^ Hormones from peripheral tissues such as leptin, ghrelin, cholecystokinin, pancreatic polypeptide, PYY (PYY3–36), GLP‐1, and oxyntomodulin have been shown to regulate appetite.^39^, ^40^, ^41^, ^42^, ^43^, ^44^, ^45^, ^46^, ^47^ Resistance to the actions of some of these hormones appears to be associated with common obesity. For example, leptin is secreted by adipose tissue and is thought to be a key peptide in reducing food intake based on the extreme obesity that develops in the absence of leptin signaling.^38^, ^48^ However, people affected by obesity have chronically elevated leptin levels and are resistant to its anorexigenic effects^39^, ^48^—this is thought to be caused, in part, by downregulation of a feedback loop by the high leptin levels.^49^ Food intake is also regulated by the mesolimbic reward system and has been shown to activate some of the same circuits involved in drug addiction.^38^, ^50^, ^51^, ^52^

The pathophysiology of obesity is complex and currently approved therapies for obesity only target a few of the many pathways involved; thus, single‐targeting agents have limited efficacy.^22^, ^53^ An integrated approach to the treatment of obesity that targets multiple mechanisms such as feeding circuits, glucose metabolism, and energy expenditure is therefore assumed to be more effective than single‐targeting agents.^53^ Proglucagon‐derived peptides, glucagon, and the incretin hormones GLP‐1 and glucose‐dependent insulinotropic polypeptide (GIP), represent attractive targets for managing obesity and metabolic disorders^53^, ^54^, ^55^, ^56^ because they may play a direct role in multiple mechanisms involved in the disease, including satiety, energy homeostasis, and lipolytic activity.^46^, ^57^, ^58^, ^59^

Dipeptidyl peptidase‐4 (DPP‐4) inhibitors, approved for use in T2DM,^60^ prevent DPP‐4 from cleaving various gut peptides including GLP‐1 and GIP^22^, ^61^; however, levels of GLP‐1 activity achieved by DPP‐4 inhibitors alone are not sufficient to stimulate a decrease in bodyweight.^22^, ^61^, ^62^ Furthermore, DPP‐4 inhibition stops the conversion of PYY 1–36 to PYY 3–36, the molecular form that reduces appetite and food intake,^63^ and this may further limit the effects on bodyweight loss because what is gained with respect to the effects of GLP‐1 (and GIP) is lost with respect to the effects of PYY.^32^

GLP‐1 has a short half‐life and is cleaved by DPP‐4 and neutral endopeptidase within 1.5–2 minutes. This has led to the development of GLP‐1R agonists that have higher enzymatic stability towards both peptidases than endogenous GLP‐1, resulting in slower elimination.^62^ However, because the peptide is also cleared by the kidneys, prolongation techniques have been developed to ensure lasting agonism. For example, the GLP‐1R agonist liraglutide is acylated and its acyl moiety (palmitic acid) binds to albumin, whereby the peptide survives in the circulation.^64^ This agonist has been shown to effectively cause bodyweight loss in humans and experimental animals, in which sufficient levels of the natural peptide do not remain in the circulation to account for this effect.^65^, ^66^, ^67^ Investigations using rat models demonstrate that liraglutide may cross the blood–brain barrier via the circumventricular organs (the area postrema, the subfornical organ, the choroid plexus, and the median eminence) and reach, for instance, the arcuate nucleus.^67^ Here, liraglutide could activate neurons expressing proopiomelanocortin (POMC) and cocaine‐ and amphetamine‐regulated transcript (CART), which are key appetite‐regulating neurons, and indirectly inhibit neurotransmission in neurons expressing neuropeptide Y (NPY) and agouti‐related peptide (AgRP) via GABA‐dependent signaling.^67^ Other long‐acting GLP‐1R agonists that target the gastrointestinal (GI) tract and central nervous system (CNS), including dulaglutide, exenatide extended‐release, and semaglutide, have since been developed and reduce bodyweight to a similar (~2%–3%) or, in the case of injectable semaglutide, greater (~4%–6%) extent as liraglutide with a similar tolerability profile in humans.^68^

As GLP‐1, GIP, and glucagon have related peptide sequences, it is possible to create analogues with agonist activity at more than one receptor type, for instance, combining GLP‐1R agonist activity with the effects of glucagon and/or GIP.^61^ Here, we discuss preclinical and clinical findings in obesity and other therapeutic areas of interest for glucagon, the endogenous incretin hormones GIP and GLP‐1 and GLP‐1R agonists, as well as their actions when combined as dual and triple agonists.

## 2 GLUCAGON IN OBESITY

**FIGURE 1:**
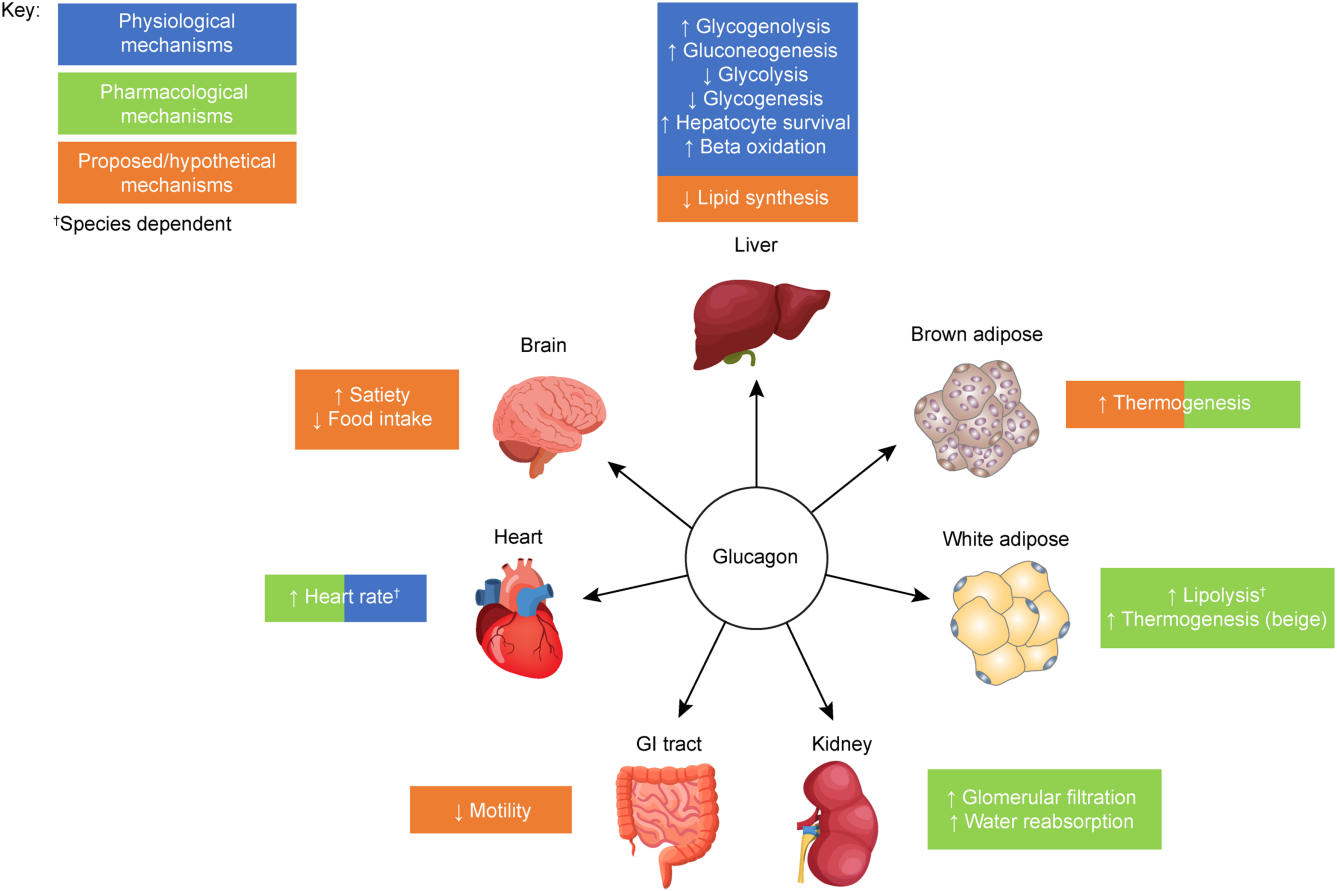
Physiological and pharmacological actions of glucagon. Glucagon has a number of physiological (blue), pharmacological (green), and hypothetical (orange) actions in several organs, some of which may be species dependent. GI, gastrointestinal

Glucagon is a pancreatic hormone, with receptors predominantly expressed in the liver. There also appear to be receptors expressed in the kidneys (although the localization is uncertain), while expression in the heart, adipose tissue, CNS, adrenal gland, and spleen is variable and may be species dependent (Figure 1).^66^

Glucagon regulates amino acid metabolism and is released from alpha cells following amino acid stimulation as part of the liver–alpha cell axis.^69^, ^70^, ^71^ In addition, glucagon has long been recognized to regulate glucose homeostasis, counteracting the actions of insulin by stimulating hepatic glucose production (glycogenolysis and gluconeogenesis).^61^ Glucagon, at least at pharmacological doses, may regulate lipid metabolism, energy expenditure, and food intake in multiple species.^54^, ^58^, ^72^, ^73^, ^74^, ^75^, ^76^ In humans, hepatic fat synthesis is suppressed after glucagon administration.^54^ Glucagon stimulates beta‐oxidation of fatty acids and inhibits the formation of malonyl‐coenzyme A, the first intermediate of fatty acid synthesis.^77^ However, the extent to which glucagon influences whole‐body lipid metabolism, particularly in individuals affected by obesity, remains controversial.^58^, ^77^ In rodents, glucagon has been shown to stimulate lipolysis in adipocytes^78^, ^79^, ^80^; however, glucagon receptor expression has not been successfully demonstrated in human adipocytes.^77^ The potential lipolytic effect of glucagon in humans has only been shown in vitro and at concentrations much higher than physiological levels in plasma.^77^ Glucagon may also increase energy expenditure by inducing thermogenesis in brown adipose tissue (BAT), as shown in humans and in animal models.^81^, ^82^, ^83^ This thermogenic effect is thought to be mediated through activity of the sympathetic nervous system, given that inhibiting β‐adrenergic activity impairs the ability of glucagon to increase energy expenditure.^84^ However, the contribution of thermogenesis to overall energy expenditure remains unknown, and this effect may be too small to result in bodyweight loss.^75^ In animal models, glucagon reduces food intake when administered peripherally and into the CNS.^56^, ^66^, ^85^, ^86^ Because of the extremely short half‐life of glucagon in rodents,^87^ long‐acting glucagon analogues are likely to be more effective. Glucagon infused into the hepatic portal vein reduces spontaneous meal size in rats.^85^ Conversely, infusion of anti‐glucagon antibodies into the hepatic portal vein increases spontaneous meal size in rats.^85^, ^88^ These observations have led to the suggestion that glucagon may act in the liver to generate a satiety signal that is relayed to the brain via the hepatic branch of the vagus nerve.^85^ Glucagon infusion at pharmacological doses in humans has been demonstrated to increase, rather than decrease, respiratory quotient and carbohydrate oxidation.^81^ However, increases in energy expenditure have been reported at doses that did not activate the sympathetic nervous system.^89^

In patients with diabetes, levels of glucagon are elevated during fasting and, in response to carbohydrate ingestion, the normal suppression is delayed or even briefly reversed. These abnormalities are important for the development of diabetic hyperglycemia, as indicated by the results of glucagon receptor (GCGR) antagonist administration, which may normalize glucose levels.^90^ However, as a therapy for T2DM, GCGR antagonists have shown undesirable AEs including elevated liver enzymes, accumulation of liver triglycerides, and hyperglucagonemia, which have discouraged further development of GCGR antagonists in this patient population.^61^ Inappropriate glucagon secretion and regulation has been shown in patients with obesity, as well as those with NASH.^71^, ^91^, ^92^, ^93^ The inappropriate elevation of circulating glucagon is likely the consequence of increased levels of plasma amino acids, representing a disruption of the liver–alpha cell axis caused by hepatic fat accumulation.^71^, ^94^ Hepatic steatosis can lead to glucagon resistance, wherein glucagon‐induced amino acid metabolism is impaired, causing elevated plasma levels amino acids and hence also glucagon.^71^ Indeed, it may be that among patients with T2DM, those with nonalcoholic fatty liver disease (the vast majority) and hyperaminoacidemia also have hyperglucagonemia.^92^ This disruption of the liver–alpha cell axis is mainly due to the accumulation of intrahepatic lipid, and may contribute to the development of T2DM, rather than being a consequence of it.^71^, ^92^

## 3 GLP‐1 IN OBESITY

**FIGURE 2:**
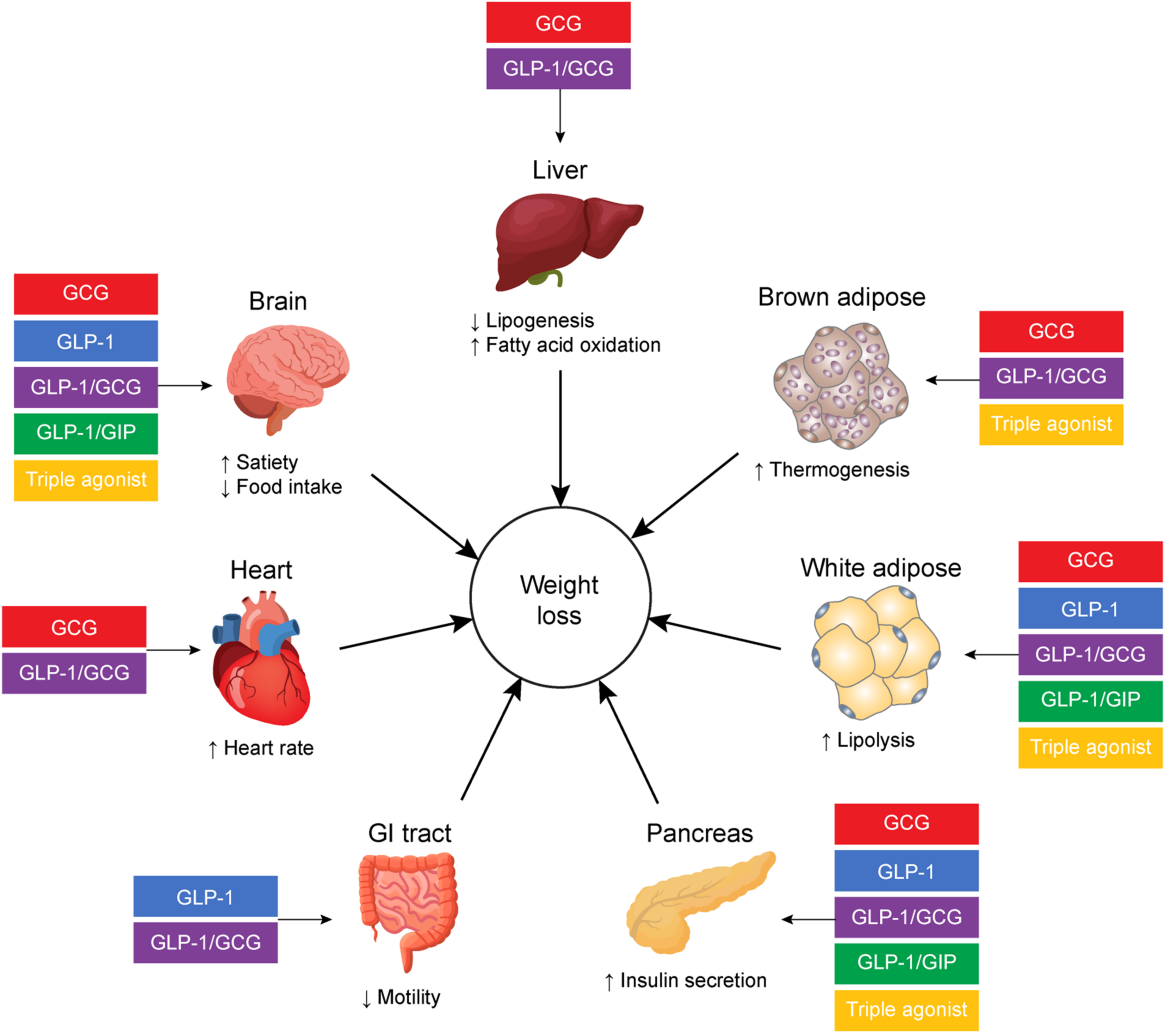
Incretin/glucagon‐targeting agents achieve their weight loss effect through a variety of mechanisms in several organs. GCG, glucagon; GI, gastrointestinal; GIP, glucose‐dependent insulinotropic polypeptide; GLP‐1, glucagon‐like peptide‐1

GLP‐1, an incretin hormone secreted from the L cells in the small intestine after food intake, stimulates insulin secretion (in a glucose‐dependent manner) and regulates energy intake.^46^, ^95^, ^96^, ^97^ GLP‐1 is also produced in the caudal portion of the nucleus of the solitary tract, a region receiving afferent input from the GI tract.^98^, ^99^ GLP‐1 acts on peripheral and central receptors in the gut and brain to delay gastric emptying, inhibit GI secretion, and decrease food intake through activation of satiety pathways and efferent pathways regulating GI function (Figure 2).^66^, ^67^, ^95^, ^100^, ^101^ GLP‐1 also reduces glucagon secretion by alpha cells, thereby inhibiting hepatic glucose production.^102^, ^103^ The GLP‐1R agonist liraglutide has been shown to reduce bodyweight in patients with prediabetes and in those with obesity,^104^ and has been approved for weight management in adults with obesity as an adjunct to a reduced‐calorie diet and increased physical activity.^65^ In addition, results from the STEP 3 trial demonstrate that the GLP‐1R agonist semaglutide reduces bodyweight in adults with obesity.^105^

## 4 GIP IN OBESITY

GIP, an incretin hormone secreted from K cells in the upper gut, acts in concert with GLP‐1 to exert “the incretin effect”, resulting in substantial physiological stimulation of insulin secretion after glucose administration.^62^, ^106^, ^107^, ^108^ In contrast to GLP‐1, GIP may stimulate glucagon secretion at lower glucose levels.^62^ Although the insulinotropic activity of GIP has now been confirmed in human studies involving a GIP receptor (GIPR) antagonist,^59^, ^109^ whether GIP contributes to the development of obesity remains controversial.^110^ Mice lacking the GIPR are protected from diet‐induced obesity, and crossing of GIPR‐null mice with obese ob/ob mice reduces adiposity.^111^, ^112^ However, other studies have demonstrated a reduction in calorie intake and bodyweight after both central and peripheral administration of GIPR agonists.^113^, ^114^ This effect is potentially mediated by GIP‐recruited neuropeptides linked to regulation of food intake and energy balance.^115^ GIP does not appear to have any acute effects on food intake in humans,^116^ yet discussions are ongoing on the role of GIPR agonists and antagonists as weight loss agents.^117^

## 5 DUAL GLP‐1R/GCGR AGONISTS

In animal models of obesity, administration of dual GLP‐1R/GCGR agonists resulted in superior weight loss, lower glucose levels, and reduced food intake compared with pure GLP‐1R agonists alone.^118^, ^119^, ^120^, ^121^ Weight loss with a dual GLP‐1R/GCGR agonist was maintained over 7 days, whereas the effect of a pure GLP‐1R agonist alone plateaued midweek before returning to vehicle control level by Day 7.^119^ In humans, dual GLP‐1R/GCGR agonism is thought to result in additive effects of reducing food intake and lowering glucose levels, making this an attractive approach for weight management in individuals with diabetes. In a Phase II trial, individuals with T2DM and who were overweight or affected by obesity treated with the dual GLP‐1R/GCGR agonist cotadutide (MEDI0382) achieved significant lowering of glucose levels and bodyweight loss compared with patients receiving placebo over 41 days (*p* < 0.0001 and *p* = 0.0008, respectively).^122^ Decreased appetite occurred more frequently in patients receiving cotadutide than those receiving placebo (20% vs. 0%); however, GI disorders were also more frequent (74% vs. 40%).^122^ Overall, the proportion of patients experiencing treatment‐emergent AEs was similar in both groups (88% vs. 88%).^122^ In a Phase IIb trial of cotadutide in patients with overweight/obesity and T2DM, significant reductions in glycated hemoglobin levels (*p* < 0.001) and percentage of bodyweight (*p* < 0.001) were observed at all tested doses (100, 200, or 300 μg) of cotadutide versus placebo, and significant reductions in the percentage of bodyweight were seen with 300‐μg cotadutide versus liraglutide (*p* = 0.009).^123^ In addition, treatment with cotadutide improved hepatic parameters, with decreases in alanine aminotransferase, aspartate aminotransferase, gamma‐glutamyl transferase, and procollagen III levels and improvements in nonalcoholic fatty liver disease fibrosis score and Fibrosis‐4 index compared with placebo, whereas liraglutide had no notable effect.^123^ The incidence of treatment‐emergent AEs was higher across all doses of cotadutide compared with placebo and liraglutide, with GI disorders being most commonly reported.^123^ In overweight individuals without diabetes, dual GLP‐1/glucagon infusion increased energy expenditure to a similar degree as glucagon alone; however, the addition of GLP‐1 reduced the hyperglycemic effect of glucagon.^124^ Dual GLP‐1/glucagon infusion has been reported to significantly reduce food intake (−13%, *p* < 0.05) compared with similar doses of GLP‐1 and glucagon administered separately, although patients reported postprandial nausea and some vomiting.^124^ A trend towards increased pulse rate was also seen with dual GLP‐1/glucagon infusion compared with placebo or GLP‐1 alone, although no substantial change in blood pressure was recorded.^81^ Thus, concomitant GCGR and GLP‐1R activation provides the beneficial effects of glucagon (i.e., maintaining a significant reduction in food intake with little effect on plasma glucose levels; Figure 2).^81^, ^124^

## 6 DUAL GLP‐1R/GIPR AGONISTS

Although the lipogenic potential of GIP alone is under debate, coactivation of GLP‐1R and GIPR is an attractive prospect in the treatment of T2DM and perhaps obesity (Figure 2).^125^ For example, GIP analogues that do not alter bodyweight when administered alone to mice with diet‐induced obesity were found to enhance GLP‐1‐induced weight loss, reduce food intake, and prevent fat mass accumulation^126^, ^127^; however, similar results have also been obtained with GIP antibodies.^128^ The dual GLP‐1R/GIPR agonist tirzepatide (LY3298176) has been shown to improve insulin sensitivity independently of GLP‐1R‐induced weight loss in *Glp‐1r*‐null mice (i.e., via GIPR antagonism), but whether this effect is present in humans remains to be seen.^129^ Furthermore, a balanced unimolecular GLP‐1R and GIPR agonist reduced bodyweight, food intake, and fat mass in mice with diet‐induced obesity to a greater extent than liraglutide.^127^ Although the exact mechanisms of GLP‐1/GIP synergism are unclear, it has been hypothesized that GIP could act directly via the CNS by inhibiting food intake, enhancing the anorexigenic action of GLP‐1, or increasing tolerability to GLP‐1R agonists.^130^ Dual GLP‐1R/GIPR agonism has also shown efficacy in humans. In a Phase II trial of tirzepatide, more individuals with T2DM achieved weight loss of ≤5% and ≤10%, and glucose control with the dual GLP‐1R/GIPR agonist than with a GLP‐1R agonist (dulaglutide) alone.^131^ Decreased appetite (although desirable) was the second most common AE, with dose‐related GI events being the most common but the majority being transient and mild to moderate in severity.^131^ In the Phase III SURPASS‐2 trial, treatment with tirzepatide was superior to semaglutide at reducing bodyweight in patients with T2DM at all tested doses (5, 10, or 15 mg), with 34%–57% of patients receiving tirzepatide experiencing bodyweight reductions of ≥10%, compared with 24% of those receiving semaglutide (1 mg).^132^ In the Phase III SURPASS‐3 trial of tirzepatide (5, 10, or 15 mg) in individuals with T2DM (with or without metformin and/or an SGLT‐2 inhibitor), bodyweight reduction ranged from −9.8 to −15.2 kg.^133^ The most commonly reported AEs in the tirzepatide arms were GI related and generally mild to moderate in severity, with up to ~11% of participants in the tirzepatide arms discontinuing treatment due to AEs.^134^

## 7 TRIPLE GLP‐1R/GCGR/GIPR AGONISTS

The synergistic actions of glucagon to reduce food intake and increase energy expenditure, GLP‐1 to reduce calorie intake, and GIP to potentiate bodyweight loss may aid in the treatment of obesity (Figure 2). The addition of both incretin components to glucagon appear to better mitigate the hyperglycemic action of glucagon compared with the presence of GLP‐1 or GIP alone, allowing for greater glucagon dosing and therefore greater potential for weight loss.^135^ In animal models of obesity, balanced unimolecular triple agonism proved superior to existing dual agonists and best‐in‐class monoagonists in reducing bodyweight and enhancing glycemic control.^136^ In a murine model of diet‐induced NASH and fibrosis, the triple combination of GLP‐1R, GCGR, and GIPR monoagonists increased bodyweight loss, reduced liver triglycerides, and improved histological NASH disease activity score; weight loss was similar to that obtained with liraglutide alone, but histological NASH disease activity score was significantly improved (*p* < 0.01).^137^ In addition, HM15211, a long‐acting triple agonist peptide, reduced bodyweight and improved liver function in cynomolgus monkey models of obesity and NASH.^138^

## 8 BALANCED AGONISM, SPECIFICITY, AND SELECTIVITY

Activation of multiple receptors can be achieved by either a combination of two or more different monoagonists or a unimolecular multiagonist. A multiagonist may take the form of a multivalent fusion of monoagonist analogues or a hybridized molecule comprising multiple epitope regions that has an overall size comparable with the native peptides.^61^ The latter approach is favored when targeting GLP‐1R, GCGR, and/or GIPR, because they are the same type of receptor (class B G‐protein coupled) and have a high degree of sequence homology and native ligands with similar secondary structures.^61^ The GCGR, GIPR, and especially GLP‐1R exhibit cross‐reactivity with each other's ligands, with glucagon being the most cross‐reactive ligand^61^; thus, a full investigation and characterization of the interactions at the relevant receptors is required. For example, LY2409021, originally developed as a GCGR antagonist, was subsequently found to block the actions of glucagon at the GCGR and GLP‐1R, the actions of GLP‐1 at the GLP‐1R, and the actions of GIP at the GIPR in vitro.^139^ When designing unimolecular dual and triple agonist peptides, it is important to consider whether the molecule activates all target receptors with equal potency (balanced agonism) or has a higher affinity for one receptor over the other(s) (preferential agonism).^61^ An appropriately balanced unimolecular agonist can only occupy a single receptor at a time, which theoretically reduces the likelihood of preferential binding at any one type of receptor, as could happen with a multivalent fusion of agonists with different affinities.^61^ In addition, the selectivity of an agonist for a given receptor has relevance for predicting and, ultimately, avoiding off‐target effects.^139^

## 9 AGENTS TARGETING THE INCRETIN/GLUCAGON SYSTEM IN OBESITY

The synergy of dual and triple incretin agonists in increasing bodyweight loss through decreased appetite and increased energy expenditure may offer an advanced therapeutic option for patients with obesity, and several novel unimolecular peptides are in clinical development (Table 2). Most trials have yet to be fully published, and the majority of published reports describe early pharmacokinetic and tolerability studies; nevertheless, trials of GG‐co‐agonist 1177, JNJ‐6456511, BI 456906, and tirzepatide are currently investigating bodyweight‐related outcomes.

**TABLE 2:** Summary of clinical trials of agents targeting the incretin/glucagon system under investigation in patients with obesity

Agonist	Agent	Trial phase	Selected outcome measures	Trial number
Single agonists				
GCGR agonist	NN9030	Phase I	PK/safety	NCT02235961
		Phase I	PK/safety; Δ HbA1c	NCT02870231
		Phase I	PK/safety; Δ HbA1c	NCT02835235
Dual agonists				
GLP‐1R/GCGR agonists	GG‐co‐agonist 1177	Phase I	PK/safety; Δ bodyweight	NCT02941042
		Phase I	PK/safety	NCT03308721
	JNJ‐6456511	Phase I	PK/safety	NCT03586843
		Phase II (T2DM)	Δ bodyweight; ≥5% bodyweight loss	NCT03586830
		Phase II	Δ bodyweight; ≥5% and ≥10% bodyweight loss	NCT03486392
	MOD 6031	Phase I	PK/safety	NCT02692781
	BI 456906	Phase I	PK/safety	NCT03591718
		Phase I	PK/safety	NCT04384081
		Phase II	Δ bodyweight; ≥5%, ≥10%, and ≥15% bodyweight loss	NCT04667377
		Phase II (T2DM)	Δ HbA1c; Δ bodyweight; ≥5% and ≥10% bodyweight loss	NCT04153929
GLP‐1R/GIPR agonists	Tirzepatide (LY3298176)	Phase I	Δ food intake; Δ EE; Δ RQ; Δ % body fat; Δ FFA; Δ postmeal glucose	NCT04081337
		Phase I	Δ energy intake; Δ appetite VAS	NCT04311411
		Phase I (±T2DM)	PK; Δ HbA1c	NCT04407234
		Phase III (T2DM)	Δ bodyweight; ≥5%, ≥10%, and ≥15% bodyweight loss; Δ WC; Δ BMI; Δ fasting glucose and insulin; Δ HbA1c; Δ lipids; Δ BP; Δ QOL	NCT04657003
		Phase III	Δ bodyweight; ≥5%, ≥10%, and ≥15% bodyweight loss; Δ WC; Δ BMI; Δ fasting glucose and insulin; Δ HbA1c; Δ lipids; Δ BP; Δ QOL	NCT04657016
		Phase III	Δ bodyweight; ≥5% and ≥10% bodyweight loss; Δ WC; Δ BMI; Δ fasting glucose and insulin; Δ HbA1c, Δ lipids; Δ BP; Δ QOL	NCT04660643
		Phase III	MACE	NCT04255433
		Phase III	Δ bodyweight; ≥5%, ≥10%, and ≥15% bodyweight loss; Δ WC; Δ BMI; Δ fasting glucose and insulin; time to T2DM onset; Δ HbA1c; Δ lipids; Δ BP; Δ QOL	NCT04184622
Triple agonists				
GLP‐1R/GCGR/GIPR agonists	Triagonist 1706	Phase I	PK/safety	NCT03095807
		Phase I	PK/safety	NCT03661879
	HM15211	Phase I	Safety	NCT03374241
		Phase I	Safety	NCT03744182

Abbreviations: BMI, body mass index; BP, blood pressure; EE, energy expenditure; FFF, free fatty acids; HbA1c, glycated hemoglobin; MACE, major adverse cardiac event; PK, pharmacokinetics; QOL, quality of life; RQ, respiratory quotient; T2DM, type 2 diabetes; VAS, visual analogue score; WC, waist circumference.

## 10 SAFETY

Glucagon and related peptides have a multitude of hormonal and metabolic effects and not all are desirable when targeting the receptors therapeutically.^66^ Some unwanted effects are usually classified as GI, although it is likely that they are mainly due to interactions with central receptors. Whereas delayed gastric emptying may be sensed as fullness, one consequence of the interaction with area postrema receptors triggered by GLP‐1 and glucagon appears to be mild‐to‐moderate transient nausea,^46^, ^124^, ^131^ which has also been reported in studies of single GLP‐1 agonists in patients with diabetes.^25^, ^104^ Additional GI AEs (vomiting and diarrhea) have been observed in trials of GLP‐1R/GCGR dual agonists.^122^, ^131^ Cardiovascular AEs are of potential concern, and a number of cardiovascular outcomes trials will be required as development continues, such as the ongoing SURPASS‐CVOT of tirzepatide.^140^ Completed trials of the GLP‐1R agonists liraglutide, semaglutide, and dulaglutide have demonstrated superiority with respect to rates of adverse cardiac outcomes in comparison with placebo.^141^, ^142^, ^143^

## 11 CONCLUSIONS

Obesity is associated with a considerable and progressive disease burden, and an effective pharmacological intervention is lacking. Glucagon is an attractive target for bodyweight management in individuals with obesity due to its ability to reduce food intake and stimulate energy expenditure, potentially without cardiovascular AEs. However, its action may need to be counterbalanced by concomitant use of incretin hormones (i.e., preventing hyperglycemia and enhancing the central effects of glucagon). The incretin hormone GLP‐1 is also an attractive target because it suppresses appetite and reduces food intake, although the role of the incretin hormone GIP in bodyweight reduction is under debate. GIPR agonism alone has been shown to reduce bodyweight in mice with obesity, as observed with GIPR agonists with a longer half‐life than endogenous GIP. However, these agents alone may have limited efficacy. It is reasonable to assume that the dual and triple combinations of glucagon and incretin hormone receptor agonists could provide superiority in maximizing bodyweight loss. Unimolecular dual and triple agonists that target glucagon and incretin hormone receptors have been shown to improve bodyweight loss, lower glucose levels, and reduce food intake in animal models of obesity and NASH, and multiple dual agonists are in clinical development for the treatment of obesity and diabetes. Phase II clinical data have established that the dual GLP‐1R/GIPR agonist tirzepatide has superior antidiabetic efficacy compared with the GLP‐1R agonist dulaglutide, alongside reductions in bodyweight and the induction of satiety. Reductions in bodyweight and glucose levels have also been demonstrated with dual GLP‐1R/GCGR agonists. The extent to which glucagon contributes to such treatment effects remains to be understood, but it may contribute to weight loss by reducing appetite and food intake, while concomitant GLP‐1R agonism ensures normal glucose control. Further research is required to fully understand the molecular mechanisms of action that underpin the efficacy of both dual and triple receptor agonists and the respective metabolic effects.

## CONFLICT OF INTEREST

SDP reports receiving grants or contracts from AstraZeneca, Boehringer Ingelheim, and Merck Sharp & Dohme; reports receiving honoraria from AstraZeneca, Boehringer Ingelheim, Eli Lilly and Co, Merck Sharp & Dohme, Novartis Pharmaceuticals, Novo Nordisk, Sanofi, and Takeda Pharmaceuticals; and has participated on Data Safety Monitoring/Advisory Boards for GlaxoSmithKline, Novartis Pharmaceuticals, Eli Lilly and Co, Sanofi, Applied Therapeutics, and Novo Nordisk. BG reports receiving consultancy fees from AstraZeneca, Bayer, Boehringer Ingelheim, Eli Lilly and Co, Merck Sharp & Dohme, Novartis, and Novo Nordisk and receiving honoraria from AstraZeneca, Bayer, Boehringer Ingelheim, Eli Lilly and Co, Bristol Myers Squibb, Merck Sharp & Dohme, and Novo Nordisk. JJH reports receiving consultancy fees from Novo Nordisk and Merck; reports receiving honoraria from Novo Nordisk and Merck; is a co‐inventor on patents covering GIPR ligands and dual‐acting GIP/GLP‐2 agonists; and is a minority shareholder and board member of Antag Therapeutics. JM reports receiving grants or contracts from Merck Sharp & Dohme and Novo Nordisk; receiving consultancy fees from AstraZeneca, Bristol Myers Squibb, Boehringer Ingelheim, Eli Lilly and Co, Merck Sharp & Dohme, Novo Nordisk, and Sanofi; receiving honoraria from AstraZeneca, Bristol Myers Squibb, Boehringer Ingelheim, Eli Lilly and Co, Merck Sharp & Dohme, Novo Nordisk, and Sanofi; and receiving support for attending meetings/travel from Novo Nordisk.

## References

[1] World Health Organization . Fact sheet on obesity and overweight. 2020. Available at: https://www.who.int/en/news-room/fact-sheets/detail/obesity-and-overweight (Accessed 12 August 2021).

[2] Bray GA , Kim KK , Wilding JPH , World Obesity F . Obesity: a chronic relapsing progressive disease process. A position statement of the World Obesity Federation. Obes Rev. 2017;18:715‐723. 10.1111/obr.12551 28489290

[3] World Health Organization (WHO) . Report of a WHO consultation on obesity. In: Obesity: preventing and managing the global epidemic. Geneva: WHO; 1998 Available at: http://whqlibdoc.who.int/hq/1998/WHO_NUT_NCD_98.1_(p1-158).pdf (Accessed 12 August 2021).

[4] Khan SS , Ning H , Wilkins JT , et al. Association of body mass index with lifetime risk of cardiovascular disease and compression of morbidity. JAMA Cardiol. 2018;3:280‐287. 10.1001/jamacardio.2018.0022 29490333 PMC5875319

[5] Guh DP , Zhang W , Bansback N , Amarsi Z , Birmingham CL , Anis AH . The incidence of co‐morbidities related to obesity and overweight: a systematic review and meta‐analysis. BMC Public Health. 2009;9:88. 10.1186/1471-2458-9-88 19320986 PMC2667420

[6] Younossi ZM . Non‐alcoholic fatty liver disease—a global public health perspective. J Hepatol. 2019;70:531‐544. 10.1016/j.jhep.2018.10.033 30414863

[7] Buratta L , Pazzagli C , Delvecchio E , Cenci G , Germani A , Mazzeschi C . Personality features in obesity. Front Psychol. 2021;11:530425. 10.3389/fpsyg.2020.530425 33519568 PMC7840523

[8] Folsom AR , Jensen MD , Jacobs DR Jr , Hilner JE , Tsai AW , Schreiner PJ . Serum leptin and weight gain over 8 years in African American and Caucasian young adults. Obes Res. 1999;7:1‐8. 10.1002/j.1550-8528.1999.tb00384.x 10023724

[9] Anderson JW , Konz EC , Frederich RC , Wood CL . Long‐term weight‐loss maintenance: a meta‐analysis of US studies. Am J Clin Nutr. 2001;74:579‐584. 10.1093/ajcn/74.5.579 11684524

[10] Stokes A , Collins JM , Grant BF , et al. Prevalence and determinants of engagement with obesity care in the United States. Obesity (Silver Spring). 2018;26:814‐818. 10.1002/oby.22173 29626388 PMC5947584

[11] Jensen MD , Ryan DH , Apovian CM , et al. 2013 AHA/ACC/TOS guideline for the management of overweight and obesity in adults: a report of the American College of Cardiology/American Heart Association Task Force on Practice Guidelines and The Obesity Society. J Am Coll Cardiol. 2014;63:2985‐3023. 10.1161/01.cir.0000437739.71477.ee 24239920

[12] Australian Department of Health National Health and Medical Research Council . Clinical practice guidelines for the management of overweight and obesity in adults, adolescents and children in Australia. 2013. Available at: https://www.nhmrc.gov.au/about-us/publications/clinical-practice-guidelines-management-overweight-and-obesity (Accessed 12 August 2021).

[13] National Institute for Health and Care Excellence . 2014 Obesity: identification, assessment and management. Clinical guideline [CG189]. November 2014. Available at: https://www.nice.org.uk/guidance/cg189 (Accessed 12 August 2021).

[14] Garvey WT , Mechanick JI , Brett EM , et al. American Association of Clinical Endocrinologists and American College of Endocrinology Comprehensive Clinical Practice Guidelines for Medical Care of Patients with Obesity. Endocr Pract. 2016;22(Suppl 3):1‐203. 10.4158/ep161356.esgl 27219496

[15] Yumuk V , Tsigos C , Fried M , et al. European guidelines for obesity management in adults. Obes Facts. 2015;8:402‐424. 10.1159/000442721 26641646 PMC5644856

[16] Apovian CM , Aronne LJ , Bessesen DH , et al. Pharmacological management of obesity: an endocrine society clinical practice guideline. J Clin Endocrinol Metab. 2015;100:342‐362. 10.1210/jc.2014-3415 25590212

[17] Shai I , Schwarzfuchs D , Henkin Y , et al. Weight loss with a low‐carbohydrate, Mediterranean, or low‐fat diet. N Engl J Med. 2008;359:229‐241. 10.1056/nejmc081747 18635428

[18] Skytte MJ , Samkani A , Petersen AD , et al. A carbohydrate‐reduced high‐protein diet improves HbA(1c) and liver fat content in weight stable participants with type 2 diabetes: a randomised controlled trial. Diabetologia. 2019;62:2066‐2078. 10.1007/s00125-019-4956-4 31338545

[19] Welton S , Minty R , O'Driscoll T , et al. Intermittent fasting and weight loss: systematic review. Can Fam Physician. 2020;66:117‐125. 32060194 PMC7021351

[20] González‐Muniesa P , Mártinez‐González M‐A , Hu FB , et al. Obesity. Nat Rev Dis Primers. 2017;3:17034. 10.1038/nrdp.2017.34 28617414

[21] National Institute of Diabetes and Digestive and Kidney Diseases . Prescription medications to treat overweight and obesity. 2016. Available at: https://www.niddk.nih.gov/health-information/weight-management/prescription-medications-treat-overweight-obesity (Accessed 12 August 2021).

[22] Williams DM , Nawaz A , Evans M . Drug therapy in obesity: a review of current and emerging treatments. Diabetes Ther. 2020;11:1199‐1216. 10.1007/s13300-020-00816-y 32297119 PMC7261312

[23] Singh AK , Singh R . Pharmacotherapy in obesity: a systematic review and meta‐analysis of randomized controlled trials of anti‐obesity drugs. Expert Rev Clin Pharmacol. 2020;13:53‐64. 10.1080/17512433.2020.1698291 31770497

[24] Sherman MM , Ungureanu S , Rey JA . Naltrexone/bupropion ER (Contrave): newly approved treatment option for chronic weight management in obese adults. Pharm Ther. 2016;41:164‐172. PMC4771085 26957883

[25] Astrup A , Carraro R , Finer N , et al. Safety, tolerability and sustained weight loss over 2 years with the once‐daily human GLP‐1 analog, liraglutide. Int J Obes. 2012;36:843‐854. 10.1038/ijo.2011.203 PMC3374073 21844879

[26] Toplak H , Ziegler O , Keller U , et al. X‐PERT: weight reduction with orlistat in obese subjects receiving a mildly or moderately reduced‐energy diet: early response to treatment predicts weight maintenance. Diabetes Obes Metab. 2005;7:699‐708. 10.1111/j.1463-1326.2005.00483.x 16219013

[27] McMahon FG , Fujioka K , Singh BN , et al. Efficacy and safety of sibutramine in obese white and African American patients with hypertension: a 1‐year, double‐blind, placebo‐controlled, multicenter trial. Arch Intern Med. 2000;160:2185‐2191. 10.1001/archinte.160.14.2185 10904462

[28] Coulter AA , Rebello CJ , Greenway FL . Centrally acting agents for obesity: past, present, and future. Drugs. 2018;78:1113‐1132. 10.1007/s40265-018-0946-y 30014268 PMC6095132

[29] Onakpoya IJ , Heneghan CJ , Aronson JK . Post‐marketing withdrawal of anti‐obesity medicinal products because of adverse drug reactions: a systematic review. BMC Med. 2016;14:191. 10.1186/s12916-016-0553-2 27894343 PMC5126837

[30] FDA requests the withdrawal of the weight‐loss drug Belviq, Belviq XR (lorcaserin) from the market. 2020. Available at: https://www.fda.gov/media/135189/download (Accessed 12 August 2021).

[31] Rubino F , Nathan DM , Eckel RH , et al. Metabolic surgery in the treatment algorithm for type 2 diabetes: a joint statement by International Diabetes Organizations. Obes Surg. 2017;27:2‐21. 10.1007/s11695-016-2457-9 27957699

[32] Svane MS , Jørgensen NB , Bojsen‐Møller KN , et al. Peptide YY and glucagon‐like peptide‐1 contribute to decreased food intake after Roux‐en‐Y gastric bypass surgery. Int J Obes. 2016;40:1699‐1706. 10.1038/ijo.2016.121 27434221

[33] Holst JJ , Madsbad S , Bojsen‐Møller KN , et al. Mechanisms in bariatric surgery: gut hormones, diabetes resolution, and weight loss. Surg Obes Relat Dis. 2018;14:708‐714. 10.1016/j.soard.2018.03.003 29776493 PMC5974695

[34] Mulla CM , Middelbeek RJW , Patti ME . Mechanisms of weight loss and improved metabolism following bariatric surgery. Ann N Y Acad Sci. 2018;1411:53‐64. 10.1111/nyas.13409 28868615 PMC5788713

[35] International Federation for the Surgery of Obesity and Metabolic Disorders . Sleeve gastrectomy. 2018. Available at: https://www.ifso.com/sleeve-gastrectomy/ (Accessed 12 August 2021).

[36] Svane MS , Bojsen‐Møller KN , Martinussen C , et al. Postprandial nutrient handling and gastrointestinal hormone secretion after Roux‐en‐Y gastric bypass vs sleeve gastrectomy. Gastroenterology. 2019;156:1627‐1641e1. 10.1053/j.gastro.2019.01.262 30742833

[37] National Institute of Diabetes and Digestive and Kidney Diseases . Bariatric surgery. 2016. Available at: https://www.niddk.nih.gov/health-information/weight-management/bariatric-surgery (Accessed 12 August 2021).

[38] Markus A . Neurobiology of obesity. Nat Neurosci. 2005;8:551. 10.1038/nn0505-551 15856060

[39] Liu J , Yang X , Yu S , Zheng R . The leptin resistance. Adv Exp Med Biol. 2018;1090:145‐163. 10.1007/978-981-13-1286-1_8 30390289

[40] Nakazato M , Murakami N , Date Y , et al. A role for ghrelin in the central regulation of feeding. Nature. 2001;409:194‐198. 10.1038/35051587 11196643

[41] Sovetkina A , Nadir R , Fung JNM , Nadjarpour A , Beddoe B . The physiological role of ghrelin in the regulation of energy and glucose homeostasis. Cureus. 2020;12:e7941. 10.7759/cureus.7941 32499981 PMC7266561

[42] Kissileff HR , Carretta JC , Geliebter A , Pi‐Sunyer FX . Cholecystokinin and stomach distension combine to reduce food intake in humans. Am J Physiol Regul Integr Comp Physiol. 2003;285:R992‐R998. 10.1152/ajpregu.00272.2003 12920059

[43] Batterham RL , Le Roux CW , Cohen MA , et al. Pancreatic polypeptide reduces appetite and food intake in humans. J Clin Endocrinol Metab. 2003;88:3989‐3992. 10.1210/jc.2003-030630 12915697

[44] Batterham RL , Cowley MA , Small CJ , et al. Gut hormone PYY(3‐36) physiologically inhibits food intake. Nature. 2002;418:650‐654. 10.1038/nature00887 12167864

[45] Turton MD , O'Shea D , Gunn I , et al. A role for glucagon‐like peptide‐1 in the central regulation of feeding. Nature. 1996;379:69‐72. 10.1038/379069a0 8538742

[46] Flint A , Raben A , Astrup A , Holst JJ . Glucagon‐like peptide 1 promotes satiety and suppresses energy intake in humans. J Clin Invest. 1998;101:515‐520. 10.1172/JCI990 9449682 PMC508592

[47] Cohen MA , Ellis SM , Le Roux CW , et al. Oxyntomodulin suppresses appetite and reduces food intake in humans. J Clin Endocrinol Metab. 2003;88:4696‐4701. 10.1210/jc.2003-030421 14557443

[48] Considine RV , Sinha MK , Heiman ML , et al. Serum immunoreactive‐leptin concentrations in normal‐weight and obese humans. N Engl J Med. 1996;334:292‐295. 10.1056/NEJM199602013340503 8532024

[49] Izquierdo AG , Crujeiras AB , Casanueva FF , Carreira MC . Leptin, obesity, and leptin resistance: where are we 25 years later? Nutrients. 2019;11. 10.3390/nu11112704 PMC6893721 31717265

[50] Subramaniapillai M , McIntyre RS . A review of the neurobiology of obesity and the available pharmacotherapies. CNS Spectr. 2017;22:29‐38. 10.1017/S1092852917000839 29350126

[51] Martel P , Fantino M . Mesolimbic dopaminergic system activity as a function of food reward: a microdialysis study. Pharmacol Biochem Behav. 1996;53:221‐226. 10.1016/0091-3057(95)00187-5 8848454

[52] Fulton S , Pissios P , Manchon RP , et al. Leptin regulation of the mesoaccumbens dopamine pathway. Neuron. 2006;51:811‐822. 10.1016/j.neuron.2006.09.006 16982425

[53] Clemmensen C , Finan B , Müller TD , DiMarchi RD , Tschöp MH , Hofmann SM . Emerging hormonal‐based combination pharmacotherapies for the treatment of metabolic diseases. Nat Rev Endocrinol. 2019;15:90‐104. 10.1038/s41574-018-0118-x 30446744

[54] Campbell JE , Drucker DJ . Islet α cells and glucagon—critical regulators of energy homeostasis. Nat Rev Endocrinol. 2015;11:329‐338. 10.1038/nrendo.2015.51 25850661

[55] Dunning BE , Foley JE , Ahrén B . Alpha cell function in health and disease: influence of glucagon‐like peptide‐1. Diabetologia. 2005;48:1700‐1713. 10.1007/s00125-005-1878-0 16132964

[56] Parker JA , McCullough KA , Field BCT , et al. Glucagon and GLP‐1 inhibit food intake and increase c‐fos expression in similar appetite regulating centres in the brainstem and amygdala. Int J Obes (2005). 2013;37:1391‐1398. 10.1038/ijo.2012.227 23337772

[57] Penick SB , Hinkle LE Jr . Depression of food intake induced in healthy subjects by glucagon. N Engl J Med. 1961;264:893‐897. 10.1056/NEJM196105042641801 13734109

[58] Pereira MJ , Thombare K , Sarsenbayeva A , et al. Direct effects of glucagon on glucose uptake and lipolysis in human adipocytes. Mol Cell Endocrinol. 2019;503:110696. 10.1016/j.mce.2019.110696 31891768

[59] Dupre J , Ross SA , Watson D , Brown JC . Stimulation of insulin secretion by gastric inhibitory polypeptide in man. J Clin Endocrinol Metab. 1973;37:826‐828. 10.1210/jcem-37-5-826 4749457

[60] Orime K , Terauchi Y . Efficacy and safety of saxagliptin for the treatment of type 2 diabetes mellitus. Expert Opin Pharmacother. 2020;1‐14. 10.1080/14656566.2020.1803280 32990096

[61] Capozzi ME , DiMarchi RD , Tschöp MH , Finan B , Campbell JE . Targeting the incretin/glucagon system with triagonists to treat diabetes. Endocr Rev. 2018;39:719‐738. 10.1210/er.2018-00117 29905825 PMC7263842

[62] Nauck MA , Meier JJ . Incretin hormones: their role in health and disease. Diabetes Obes Metab. 2018;20(Suppl 1):5‐21. 10.1111/dom.13129 29364588

[63] Unniappan S , McIntosh CH , Demuth HU , Heiser U , Wolf R , Kieffer TJ . Effects of dipeptidyl peptidase IV on the satiety actions of peptide YY. Diabetologia. 2006;49:1915‐1923. 10.1007/s00125-006-0310-8 16802131

[64] Frederiksen TM , Sønderby P , Ryberg LA , et al. Oligomerization of a glucagon‐like peptide 1 analog: bridging experiment and simulations. Biophys J. 2015;109:1202‐1213. 10.1016/j.bpj.2015.07.051 26340816 PMC4576320

[65] Liraglutide (Saxenda) Prescribing Information. Novo Nordisk A/S; 2020.

[66] Sandoval DA , D'Alessio DA . Physiology of proglucagon peptides: role of glucagon and GLP‐1 in health and disease. Physiol Rev. 2015;95:513‐548. 10.1152/physrev.00013.2014 25834231

[67] Secher A , Jelsing J , Baquero AF , et al. The arcuate nucleus mediates GLP‐1 receptor agonist liraglutide‐dependent weight loss. J Clin Invest. 2014;124:4473‐4488. 10.1172/JCI75276 25202980 PMC4215190

[68] Chun JH , Butts A . Long‐acting GLP‐1RAs: an overview of efficacy, safety, and their role in type 2 diabetes management. JAAPA. 2020;33:3‐18. 10.1097/01.JAA.0000669456.13763.bd 32740121

[69] Boden G , Rezvani I , Owen OE . Effects of glucagon on plasma amino acids. J Clin Invest. 1984;73:785‐793. 10.1172/JCI111272 6142902 PMC425081

[70] Hayashi Y , Seino Y . Regulation of amino acid metabolism and α‐cell proliferation by glucagon. J Diabetes Investig. 2018;9:464‐472. 10.1111/jdi.12797 PMC5934249 29314731

[71] Winther‐Sørensen M , Galsgaard KD , Santos A , et al. Glucagon acutely regulates hepatic amino acid catabolism and the effect may be disturbed by steatosis. Mol Metab. 2020;42:101080. 10.1016/j.molmet.2020.101080 32937194 PMC7560169

[72] Geary N , Smith GP . Pancreatic glucagon and postprandial satiety in the rat. Physiol Behav. 1982;28:313‐322. 10.1016/0031-9384(82)90081-6 7079345

[73] Geary N , Kissileff HR , Pi‐Sunyer FX , Hinton V . Individual, but not simultaneous, glucagon and cholecystokinin infusions inhibit feeding in men. Am J Physiol. 1992;262:R975‐R980. 10.1152/ajpregu.1992.262.6.R975 1621876

[74] Jiang G , Zhang BB . Glucagon and regulation of glucose metabolism. Am J Physiol Endocrinol Metab. 2003;284:E671‐E678. 10.1152/ajpendo.00492.2002 12626323

[75] Bagger JI , Holst JJ , Hartmann B , Andersen B , Knop FK , Vilsbøll T . Effect of oxyntomodulin, glucagon, GLP‐1, and combined glucagon +GLP‐1 infusion on food intake, appetite, and resting energy expenditure. J Clin Endocrinol Metab. 2015;100:4541‐4552. 10.1210/jc.2015-2335 26445112

[76] Holst JJ . Glucagon in obesity. In: Lefebvre PJ , ed. Glucagon II. Berlin, Heidelberg: Springer Berlin Heidelberg; 1983:507‐521.

[77] Galsgaard KD , Pedersen J , Knop FK , Holst JJ , Wewer Albrechtsen NJ . Glucagon receptor signaling and lipid metabolism. Front Physiol. 2019;10:413. 10.3389/fphys.2019.00413 31068828 PMC6491692

[78] Vaughan M , Steinberg D . Effect of hormones on lipolysis and esterification of free fatty acids during incubation of adipose tissue *in vitro* . J Lipid Res. 1963;4:193‐199. 10.1016/S0022-2275(20)40346-3 14168151

[79] Prigge WF , Grande F . Effects of glucagon, epinephrine and insulin on in vitro lipolysis of adipose tissue from mammals and birds. Comp Biochem Physiol B. 1971;39:69‐82. 10.1016/0305-0491(71)90254-9 5570026

[80] Livingston JN , Cuatrecasas P , Lockwood DH . Studies of glucagon resistance in large rat adipocytes: ^125^I‐labeled glucagon binding and lipolytic capacity. J Lipid Res. 1974;15:26‐32. 10.1016/S0022-2275(20)36828-0 4359539

[81] Tan TM , Field BCT , McCullough KA , et al. Coadministration of glucagon‐like peptide‐1 during glucagon infusion in humans results in increased energy expenditure and amelioration of hyperglycemia. Diabetes. 2013;62:1131‐1138. 10.2337/db12-0797 23248172 PMC3609580

[82] Billington CJ , Bartness TJ , Briggs J , Levine AS , Morley JE . Glucagon stimulation of brown adipose tissue growth and thermogenesis. Am J Physiol. 1987;252:R160‐R165. 10.1152/ajpregu.1987.252.1.R160 3028165

[83] Billington CJ , Briggs JE , Link JG , Levine AS . Glucagon in physiological concentrations stimulates brown fat thermogenesis in vivo. Am J Physiol. 1991;261:R501‐R507. 10.1152/ajpregu.1991.261.2.R501 1877708

[84] Kinoshita K , Ozaki N , Takagi Y , Murata Y , Oshida Y , Hayashi Y . Glucagon is essential for adaptive thermogenesis in brown adipose tissue. Endocrinology. 2014;155:3484‐3492. 10.1210/en.2014-1175 24949663

[85] Geary N , Le Sauter J , Noh U . Glucagon acts in the liver to control spontaneous meal size in rats. Am J Physiol. 1993;264:R116‐R122. 10.1152/ajpregu.1993.264.1.R116 8430871

[86] Kurose Y , Kamisoyama H , Honda K , et al. Effects of central administration of glucagon on feed intake and endocrine responses in sheep. Anim Sci J. 2009;80:686‐690. 10.1111/j.1740-0929.2009.00685.x 20163659

[87] Wewer Albrechtsen NJ , Kuhre RE , Windeløv JA , et al. Dynamics of glucagon secretion in mice and rats revealed using a validated sandwich ELISA for small sample volumes. Am J Physiol Endocrinol Metab. 2016;311:E302‐E309. 10.1152/ajpendo.00119.2016 27245336

[88] Le Sauter J , Noh U , Geary N . Hepatic portal infusion of glucagon antibodies increases spontaneous meal size in rats. Am J Phys. 1991;261:R162‐R165. 10.1152/ajpregu.1991.261.1.R162 1858943

[89] Salem V , Izzi‐Engbeaya C , Coello C , et al. Glucagon increases energy expenditure independently of brown adipose tissue activation in humans. Diabetes Obes Metab. 2016;18:72‐81. 10.1111/dom.12585 26434748 PMC4710848

[90] Vajda EG , Logan D , Lasseter K , et al. Pharmacokinetics and pharmacodynamics of single and multiple doses of the glucagon receptor antagonist LGD‐6972 in healthy subjects and subjects with type 2 diabetes mellitus. Diabetes Obes Metab. 2017;19:24‐32. 10.1111/dom.12752 27501510 PMC5215471

[91] Stern JH , Smith GI , Chen S , Unger RH , Klein S , Scherer PE . Obesity dysregulates fasting‐induced changes in glucagon secretion. J Endocrinol. 2019;243:149‐160. 10.1530/JOE-19-0201 31454790 PMC6994388

[92] Wewer Albrechtsen NJ , Pedersen J , Galsgaard KD , et al. The liver‐α‐cell axis and type 2 diabetes. Endocr Rev. 2019;40:1353‐1366. 10.1210/er.2018-00251 30920583

[93] Knop FK , Aaboe K , Vilsbøll T , et al. Impaired incretin effect and fasting hyperglucagonaemia characterizing type 2 diabetic subjects are early signs of dysmetabolism in obesity. Diabetes Obes Metab. 2012;14:500‐510. 10.1111/j.1463-1326.2011.01549.x 22171657

[94] Pedersen JS , Rygg MO , Kristiansen VB , et al. Nonalcoholic fatty liver disease impairs the liver‐alpha cell axis independent of hepatic inflammation and fibrosis. Hepatol Commun. 2020;4:1610‐1623. 10.1002/hep4.1562 33163832 PMC7603528

[95] Holst JJ . Long‐acting glucagon‐like peptide‐1 receptor agonist‐status December 2018. Ann Transl Med. 2019;7:83‐83. 10.21037/atm.2019.01.09 31019933 PMC6462657

[96] Mojsov S , Heinrich G , Wilson IB , Ravazzola M , Orci L , Habener JF . Preproglucagon gene expression in pancreas and intestine diversifies at the level of post‐translational processing. J Biol Chem. 1986;261:11880‐11889. 10.1016/S0021-9258(18)67324-7 3528148

[97] Mojsov S , Weir GC , Habener JF . Insulinotropin: glucagon‐like peptide I (7‐37) co‐encoded in the glucagon gene is a potent stimulator of insulin release in the perfused rat pancreas. J Clin Invest. 1987;79:616‐619. 10.1172/JCI112855 3543057 PMC424143

[98] Zheng H , Cai L , Rinaman L . Distribution of glucagon‐like peptide 1‐immunopositive neurons in human caudal medulla. Brain Struct Funct. 2015;220:1213‐1219. 10.1007/s00429-014-0714-z 24510283 PMC4127167

[99] Tang‐Christensen M , Vrang N , Larsen PJ . Glucagon‐like peptide containing pathways in the regulation of feeding behaviour. Int J Obes Relat Metab Disord. 2001;25(Suppl 5):S42‐S47. 10.1038/sj.ijo.0801912 11840214

[100] Orskov L , Holst JJ , Møller J , et al. GLP‐1 does not not acutely affect insulin sensitivity in healthy man. Diabetologia. 1996;39:1227‐1232. 10.1007/BF02658511 8897012

[101] Shah M , Vella A . Effects of GLP‐1 on appetite and weight. Rev Endocr Metab Disord. 2014;15:181‐187. 10.1007/s11154-014-9289-5 24811133 PMC4119845

[102] Ramracheya R , Chapman C , Chibalina M , et al. GLP‐1 suppresses glucagon secretion in human pancreatic alpha‐cells by inhibition of P/Q‐type Ca^2+^ channels. Physiol Rep. 2018;6:e13852. 10.14814/phy2.13852 30187652 PMC6125244

[103] Mighiu PI , Yue JT , Filippi BM , et al. Hypothalamic glucagon signaling inhibits hepatic glucose production. Nat Med. 2013;19:766‐772. 10.1038/nm.3115 23685839

[104] le Roux CW , Astrup A , Fujioka K , et al. 3 years of liraglutide versus placebo for type 2 diabetes risk reduction and weight management in individuals with prediabetes: a randomised, double‐blind trial. Lancet. 2017;389:1399‐1409. 10.1016/S0140-6736(17)30069-7 28237263

[105] Wadden TA , Bailey TS , Billings LK , et al. Effect of subcutaneous semaglutide vs placebo as an adjunct to intensive behavioral therapy on body weight in adults with overweight or obesity: the STEP 3 randomized clinical trial. JAMA. 2021;325(14):1409‐1413. 10.1001/jama.2021.1831 PMC7905697 33625476

[106] Gasbjerg LS , Helsted MM , Hartmann B , et al. GIP and GLP‐1 receptor antagonism during a meal in healthy individuals. J Clin Endocrinol Metab. 2020;105:e725‐e738. 10.1210/clinem/dgz175 32077470

[107] Sonne DP , Rehfeld JF , Holst JJ , Vilsbøll T , Knop FK . Postprandial gallbladder emptying in patients with type 2 diabetes: potential implications for bile‐induced secretion of glucagon‐like peptide 1. Eur J Endocrinol. 2014;171:407‐419. 10.1530/EJE-14-0309 24986531

[108] Gasbjerg LS , Bari EJ , Stensen S , et al. Dose‐dependent efficacy of the glucose‐dependent insulinotropic polypeptide (GIP) receptor antagonist GIP(3‐30)NH_2_ on GIP actions in humans. Diabetes Obes Metab. 2021;23:68‐74. 10.1111/dom.14186 32886401

[109] Gasbjerg LS , Helsted MM , Hartmann B , et al. Separate and combined glucometabolic effects of endogenous glucose‐dependent insulinotropic polypeptide and glucagon‐like peptide 1 in healthy individuals. Diabetes. 2019;68:906‐917. 10.2337/db18-1123 30626611

[110] Marks V , James W , Parker S . In: James WPT , Parker SW , eds. Current Approaches Obesity. Duphar Medical Relations; 1988:13‐20.

[111] Miyawaki K , Yamada Y , Ban N , et al. Inhibition of gastric inhibitory polypeptide signaling prevents obesity. Nat Med. 2002;8:738‐742. 10.1038/nm727 12068290

[112] Adriaenssens AE , Biggs EK , Darwish T , et al. Glucose‐dependent insulinotropic polypeptide receptor‐expressing cells in the hypothalamus regulate food intake. Cell Metab. 2019;30:987‐996.e986. 10.1016/j.cmet.2019.07.013 31447324 PMC6838660

[113] Mroz PA , Finan B , Gelfanov V , et al. Optimized GIP analogs promote body weight lowering in mice through GIPR agonism not antagonism. Mol Metab. 2019;20:51‐62. 10.1016/j.molmet.2018.12.001 30578168 PMC6358549

[114] NamKoong C , Kim MS , Jang BT , Lee YH , Cho YM , Choi HJ . Central administration of GLP‐1 and GIP decreases feeding in mice. Biochem Biophys Res Commun. 2017;490:247‐252. 10.1016/j.bbrc.2017.06.031 28610922

[115] Ambati S , Duan J , Hartzell DL , Choi YH , Della‐Fera MA , Baile CA . GIP‐dependent expression of hypothalamic genes. Physiol Res. 2011;60:941‐950. 10.33549/physiolres.932151 21995902

[116] Bergmann NC , Gasbjerg LS , Heimbürger SM , et al. No acute effects of exogenous glucose‐dependent insulinotropic polypeptide on energy intake, appetite, or energy expenditure when added to treatment with a long‐acting glucagon‐like peptide 1 receptor agonist in men with type 2 diabetes. Diabetes Care. 2020;43:588‐596. 10.2337/dc19-0578 31949084

[117] Holst JJ , Rosenkilde MM . GIP as a therapeutic target in diabetes and obesity: insight from incretin co‐agonists. J Clin Endocrinol Metab. 2020;105:e2710‐e2716. 10.1210/clinem/dgaa327 PMC7308078 32459834

[118] Day JW , Gelfanov V , Smiley D , et al. Optimization of co‐agonism at GLP‐1 and glucagon receptors to safely maximize weight reduction in DIO‐rodents. Biopolymers. 2012;98:443‐450. 10.1002/bip.22072 23203689

[119] Day JW , Ottaway N , Patterson JT , et al. A new glucagon and GLP‐1 co‐agonist eliminates obesity in rodents. Nat Chem Biol. 2009;5:749‐757. 10.1038/nchembio.209 19597507

[120] Pocai A , Carrington PE , Adams JR , et al. Glucagon‐like peptide 1/glucagon receptor dual agonism reverses obesity in mice. Diabetes. 2009;58:2258‐2266. 10.2337/db09-0278 19602537 PMC2750209

[121] Elvert R , Herling AW , Bossart M , et al. Running on mixed fuel‐dual agonistic approach of GLP‐1 and GCG receptors leads to beneficial impact on body weight and blood glucose control: a comparative study between mice and non‐human primates. Diabetes Obes Metab. 2018;20:1836‐1851. 10.1111/dom.13212 29938884 PMC6055720

[122] Ambery P , Parker VE , Stumvoll M , et al. MEDI0382, a GLP‐1 and glucagon receptor dual agonist, in obese or overweight patients with type 2 diabetes: a randomised, controlled, double‐blind, ascending dose and phase 2a study. Lancet. 2018;391:2607‐2618. 10.1016/S0140-6736(18)30726-8 29945727

[123] Nahra R , Wang T , Gadde KM , et al. Effects of cotadutide on metabolic and hepatic parameters in adults with overweight or obesity and type 2 diabetes: a 54‐week randomized phase 2b study. Diabetes Care. 2021;44:1433‐1442. 10.2337/dc20-2151 34016612 PMC8247525

[124] Cegla J , Troke RC , Jones B , et al. Coinfusion of low‐dose GLP‐1 and glucagon in man results in a reduction in food intake. Diabetes. 2014;63:3711‐3720. 10.2337/db14-0242 24939425

[125] Mathiesen DS , Bagger JI , Bergmann NC , et al. The effects of dual GLP‐1/GIP receptor agonism on glucagon secretion—a review. Int J Mol Sci. 2019;20:4092. 10.3390/ijms20174092 PMC6747202 31443356

[126] Nørregaard PK , Deryabina MA , Tofteng Shelton P , et al. A novel GIP analogue, ZP4165, enhances glucagon‐like peptide‐1‐induced body weight loss and improves glycaemic control in rodents. Diabetes Obes Metab. 2018;20:60‐68. 10.1111/dom.13034 28598027

[127] Finan B , Ma T , Ottaway N , et al. Unimolecular dual incretins maximize metabolic benefits in rodents, monkeys, and humans. Sci Transl Med. 2013;5:209ra151. 10.1126/scitranslmed.3007218 24174327

[128] Killion EA , Wang J , Yie J , et al. Anti‐obesity effects of GIPR antagonists alone and in combination with GLP‐1R agonists in preclinical models. Sci Transl Med. 2018;10:eaat3392. 10.1126/scitranslmed.aat3392 30567927

[129] Samms RJ , Christe ME , Collins KA , et al. GIPR agonism mediates weight‐independent insulin sensitization by tirzepatide in obese mice. J Clin Invest. 2021;131:e146353. 10.1172/JCI146353 PMC8203452 34003802

[130] Samms RJ , Coghlan MP , Sloop KW . How may GIP enhance the therapeutic efficacy of GLP‐1? Trends Endocrinol Metab. 2020;31:410‐421. 10.1016/j.tem.2020.02.006 32396843

[131] Frias JP , Nauck MA , Van J , et al. Efficacy and safety of LY3298176, a novel dual GIP and GLP‐1 receptor agonist, in patients with type 2 diabetes: a randomised, placebo‐controlled and active comparator‐controlled phase 2 trial. Lancet. 2018;392:2180‐2193. 10.1016/S0140-6736(18)32260-8 30293770

[132] Frías JP , Davies MJ , Rosenstock J , et al. Tirzepatide versus semaglutide once weekly in patients with type 2 diabetes. N Engl J Med. 2021;385:503‐515. 10.1056/NEJMoa2107519 34170647

[133] Ludvik B , Giorgino F , Jódar E , et al. Once‐weekly tirzepatide versus once‐daily insulin degludec as add‐on to metformin with or without SGLT2 inhibitors in patients with type 2 diabetes (SURPASS‐3): a randomised, open‐label, parallel‐group, phase 3 trial. Lancet. 2021;14:583‐598. 10.1016/S0140-6736(21)01443-4 34370970

[134] Tirzepatide significantly reduced A1C and body weight in people with type 2 diabetes in two phase 3 trials from Lilly's SURPASS program. 2021. Available at: https://investor.lilly.com/news-releases/news-release-details/tirzepatide-significantly-reduced-a1c-and-body-weight-people (Accessed 12 August 2021).

[135] Knerr PJ , Mowery SA , Finan B , Perez‐Tilve D , Tschöp MH , DiMarchi RD . Selection and progression of unimolecular agonists at the GIP, GLP‐1, and glucagon receptors as drug candidates. Peptides. 2020;125:170225. 10.1016/j.peptides.2019.170225 31786282

[136] Finan B , Yang B , Ottaway N , et al. A rationally designed monomeric peptide triagonist corrects obesity and diabetes in rodents. Nat Med. 2015;21:27‐36. 10.1038/nm.3761 25485909

[137] Kannt A , Madsen AN , Kammermeier C , et al. Incretin combination therapy for the treatment of non‐alcoholic steatohepatitis. Diabetes Obes Metab. 2020;22:1328‐1338. 10.1111/dom.14035 32196896

[138] Kim JK . Therapeutic effect of a novel long‐acting GLP‐1/GIP/Glucagon triple agonist (HM15211) in NASH and fibrosis animal models. Paper presented at: EASD annual meeting; October, 2018; Berlin, Germany

[139] Chepurny OG , Matsoukas MT , Liapakis G , et al. Nonconventional glucagon and GLP‐1 receptor agonist and antagonist interplay at the GLP‐1 receptor revealed in high‐throughput FRET assays for cAMP. J Biol Chem. 2019;294:3514‐3531. 10.1074/jbc.AAC119.009193 30622136 PMC6416420

[140] NCT04255433 . The effect of tirzepatide versus dulaglutide on major adverse cardiovascular events in patients with type 2 diabetes (SURPASS‐CVOT). Available at: https://clinicaltrials.gov/ct2/show/NCT04255433 (Accessed 12 August 2021).

[141] Marso SP , Daniels GH , Brown‐Frandsen K , et al. Liraglutide and cardiovascular outcomes in type 2 diabetes. N Engl J Med. 2016;375:311‐322. 10.1056/NEJMoa1607141 27295427 PMC4985288

[142] Marso SP , Bain SC , Consoli A , et al. Semaglutide and cardiovascular outcomes in patients with type 2 diabetes. N Engl J Med. 2016;375:1834‐1844. 10.1056/NEJMoa1607141 27633186

[143] Gerstein HC , Colhoun HM , Dagenais GR , et al. Dulaglutide and cardiovascular outcomes in type 2 diabetes (REWIND): a double‐blind, randomised placebo‐controlled trial. Lancet. 2019;394:121‐130. 10.1016/S0140-6736(19)31150-X 31189511

